# Computed tomographic analysis of the morphometrics and dynamics of the tricuspid annulus in secondary functional tricuspid regurgitation

**DOI:** 10.1016/j.xjon.2023.07.025

**Published:** 2023-08-25

**Authors:** Jérôme Jouan, Damian Craiem, Gilles Soulat, Virginie Bliah, Ignacio Masari, Elie Mousseaux

**Affiliations:** a Department of Cardio-thoracic Surgery, CHU Dupuytren, University of Limoges, Limoges, France; b Université Paris Cité, Paris, France; c Instituto de Medicina Traslacional, Trasplante y Bioingeniería, Favaloro University, CONICET, Buenos Aires, Argentina; d Department of Radiology, Georges Pompidou European Hospital, Paris, France

**Keywords:** tricuspid valve, cardiac CT, functional tricuspid regurgitation

## Abstract

**Objectives:**

Secondary functional tricuspid regurgitation (FTR) management remains controversial mainly due to the lack of knowledge in its pathogenesis and the difficulties to measure the actual dimensions of tricuspid annulus (TA) with current imaging methods. Using a novel method based on multiphase cardiac computed tomography (CT) scan acquisition to accurately analyze the right atrioventricular junction size, we sought to explore modifications of TA morphometry and dynamics in secondary FTR.

**Methods:**

Echocardiographic and cardiac CT studies were obtained from 21 patients with severe mitral regurgitation (MR group) and 21 patients with dilated cardiomyopathy (DCMP group). Using an in-house software, a 3-dimensiontal (3D) semiautomated delineation of the TA perimeter was assessed. Modifications of diameters, 2-dimensional/3D areas and perimeters were analyzed through time. These 2 groups of patients were compared with 30 healthy subjects, considering the presence of a significant (≥2+) versus nonsignificant (<2+) FTR in each group.

**Results:**

Maximum TA 3D areas were 7.0 ± 1.2 cm^2^/m^2^ in healthy subjects at mid-to-late diastole and were smaller than in the MR group (9.8 ± 2.1 cm^2^/m^2^, *P* < .001) and the DCMP group (9.2 ± 3.0 cm^2^/m^2^, *P* < .001). In the MR group, patients with FTR <2+ had also larger TA areas and diameters than healthy patients (*P* < .01 for all 3D/2-dimensional parameters). TA shape was more circular only in the DCMP group with FTR ≥2+ compared with other patients (*P* < .05 for eccentricity). In multivariate analysis, both RA area (*P* < .001) and RV volume (*P* = .002) were independently related to TA dilatation.

**Conclusions:**

Based on multiphase CT image analyses, TA dilatation was directly related to RV and RA enlargement. Patients with severe mitral myxomatous disease and nondysfunctional tricuspid valve had yet dilated TA, which questioned the current cut-off recommendation for concomitant tricuspid annuloplasty in this specific population.

## Graphical Abstract

**Figure:**
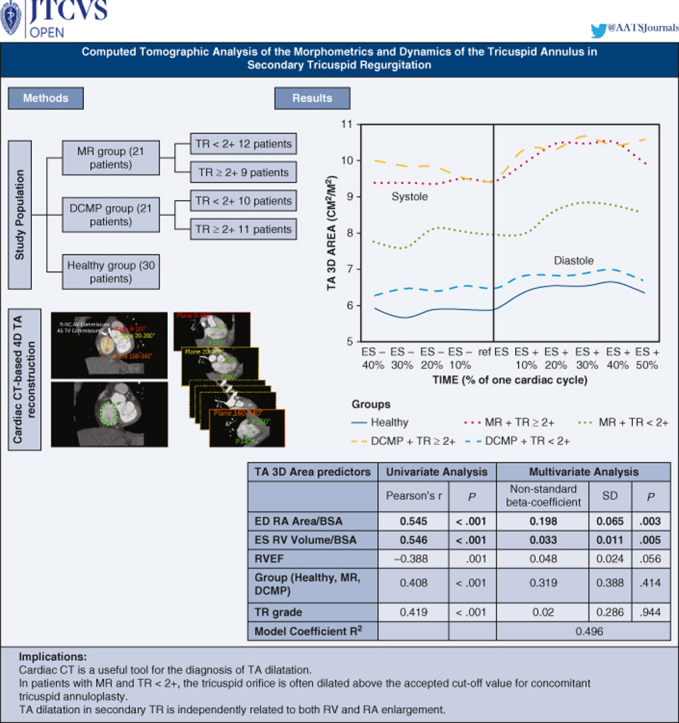

**Figure:**
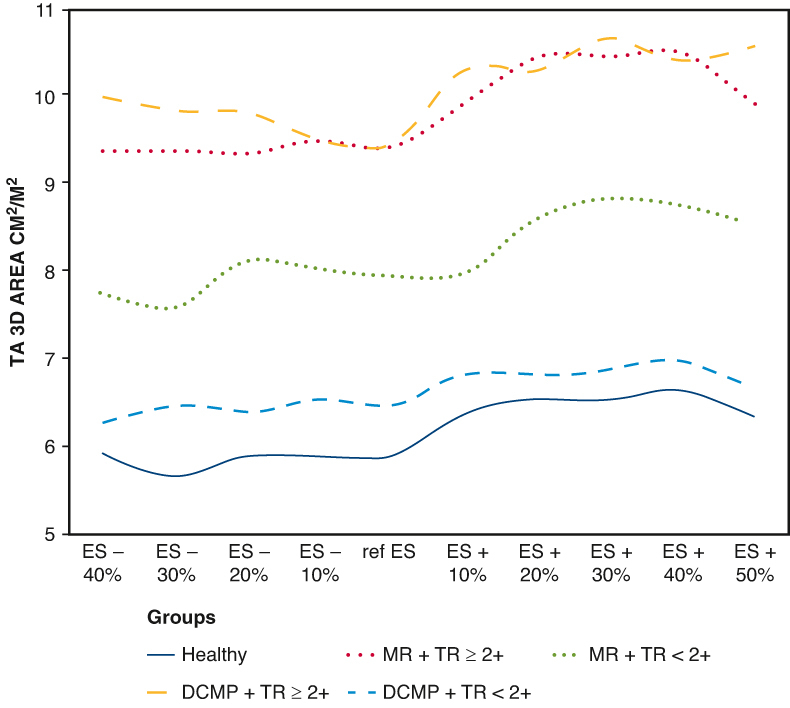
Tricuspid annulus 3D area/BSA according to groups and TR grade during the cardiac cycle.

**Central Message**

Tricuspid annulus dilatation in secondary FTR depends on both right atrium and right ventricular enlargement.

**Perspective**

Cardiac CT is a useful tool to accurately measure the 2D projected area or 3D area of the tricuspid orifice, and the dilation of the right cavities that cause tricuspid annulus dilatation. It could help over echocardiography in the decision-making process of tricuspid annuloplasty concomitant to mitral degenerative disease correction.

Functional tricuspid regurgitation (FTR) is the most common cause of tricuspid valve (TV) dysfunction.^1^^,^^2^ FTR is characterized by valve regurgitation, in the absence of leaflets/chordae damages, due to tricuspid annulus (TA) dilatation and/or right ventricle (RV) enlargement and thus functionally corresponds to either type I or type IIIb of Carpentier classification.^3^ FTR has recently been divided in 2 types: (1) isolated FTR, mainly occurring in the setting of atrial fibrillation and atrial enlargement, and (2) secondary FTR, mainly related to RV dilatation and increased afterload.^4^ Secondary FTR occurs in up to 30% of patients with severe mitral regurgitation (MR)^5^^,^^6^ and frequently increases even after successful correction of the mitral valve. TA dilatation is considered as the better predictor for worsening tricuspid regurgitation (TR) after left heart valve surgery than the degree of FTR itself.^7^^,^^8^ Since late significant secondary FTR is associated with increased morbidity and mortality, a more aggressive surgical approach toward correction of annular dilatation with or without significant FTR has been recommended at the time of left-sided heart surgery.^9^^,^^10^ For current guidelines at the time of left valve surgery, a TA diameter >40 mm or >21 mm/m^2^ obtained from echocardiographic 4-chamber view at end-diastole has to be considered for concomitant TV annuloplasty.^11^^,^^12^ Still, this approach remains controversial, particularly in mitral myxoid valve disease, where some authors report very small incidence of significant FTR late after MV valvuloplasty.^13^ Furthermore, the time-varying and frequently asymmetric geometry of the TA makes the assessment of TA size complex with 2D echocardiography. Moreover, the latter technique is not appropriate to analyze the actual size of right cavities, which could be potential determinants of TA dimensions. In order to explore the specific aspects of TA 3-dimensional (3D) geometry and dynamics in patients with various degrees of TA dilatation and secondary FTR, we conducted a comparative study on patients addressed for severe MR or dilated cardiomyopathy (DCMP) using 4-dimensional multiphase cardiac computed tomography (CT), which has been shown to be reliable for TA morphometric and dynamic analyses.^14^ The analysis of the determinants of TA dilation in this population was the secondary objective of this study.

## Methods

The institutional ethics committee of the Assistance Publique des Hôpitaux de Paris approved the prospective analysis of clinically acquired data (institutional review board #00001072-IDFII_2015, April, 2015), and the need for written informed consent was waived since all the procedures performed were part of routine care and because the included patients gave their consent for their medical data to be used for research purposes.

### Patient Population

Subjects were selected among all patients who underwent an echocardiogram and a multiphase cardiac CT at our institution for detecting coronary artery disease before cardiac surgery for severe MR due to myxomatous MV degeneration (Barlow disease) between January 2017 and June 2019. Eight patients with a bad right-side opacification or atrial fibrillation were excluded, and finally 21 patients were recruited for this study (MR group). During the same period, 21 consecutive patients in sinus rhythm were also selected with DCMP (left ventricular ejection fraction <40%) undergoing cardiac CT as part of routine assessment of the disease (DCMP group). Reported etiology of DCMP was ischemic in 9 patients, toxic/metabolic in 4, and idiopathic in 8 patients. The 42 patients with MR or DCMP were divided in 2 subgroups according to the FTR grade (<2+ and ≥2+). A total of 20 patients had ≥2+ FTR (9 with severe MR and 11 with DCMP). Furthermore, 30 patients in sinus rhythm were retrospectively selected as case-controls (healthy group). These patients have had a recent echocardiogram showing normal systolic myocardial function and no structural heart anomaly (notably absence or trace of tricuspid insufficiency), had a CT scan performed for atypical chest pain and coronary screening, and the later was considered normal. Upon admission, all subjects included in the present study signed a document consenting to the use of all data from their medical records for research purposes and scientific publications, including those from cardiac images.

### Transthoracic Echocardiography (TTE)

All selected patients having CT had 2-dimenstional (2D) echocardiography within 48 hours, including continuous, pulsed, and color Doppler performed by an experienced cardiologist with commercially available ultrasound system (Vivid-7, General Electric Vingmed, Horton, Norway, or EPIQ CVx; Phillips) at our institution. Severity of TR was evaluated using a multiparametric approach.^15^ TTE diameter was measured at the TA at end-diastole from an apical 4-chamber view.

### Cardiac CT Protocol

All CT examinations were performed on a 192-slice Dual Source CT system (SOMATOM Force; Siemens Medical Solutions) with a collimation of 2 × 192 × 0.6 mm and a temporal resolution of 66 milliseconds. Tube voltage was selected semiautomatically. Cardiac CT started by continuous injection of a bolus of 80 mL of iomeprol 350 mg/mL, followed by 30 mL of saline solution into an antecubital vein via an 18-gauge catheter (injection rate 3-5 mL/s). Retrospective electrocardiogram-gated acquisition was performed from the level of the carina to the apex of the heart in a craniocaudal direction. Electrocardiography-based tube current modulation was applied in all patients for a reduced radiation dose. Images were reconstructed at 10% to 100% of the R-R interval in 10% increments. End-systole (ES) was identified as the phase of aortic valve closing and end-diastole (ED) as the phase of mitral valve closing. Temporal phases were expressed as a percentage of the RR interval relative to the ES phase.

### TA Segmentation

**Figure 1:**
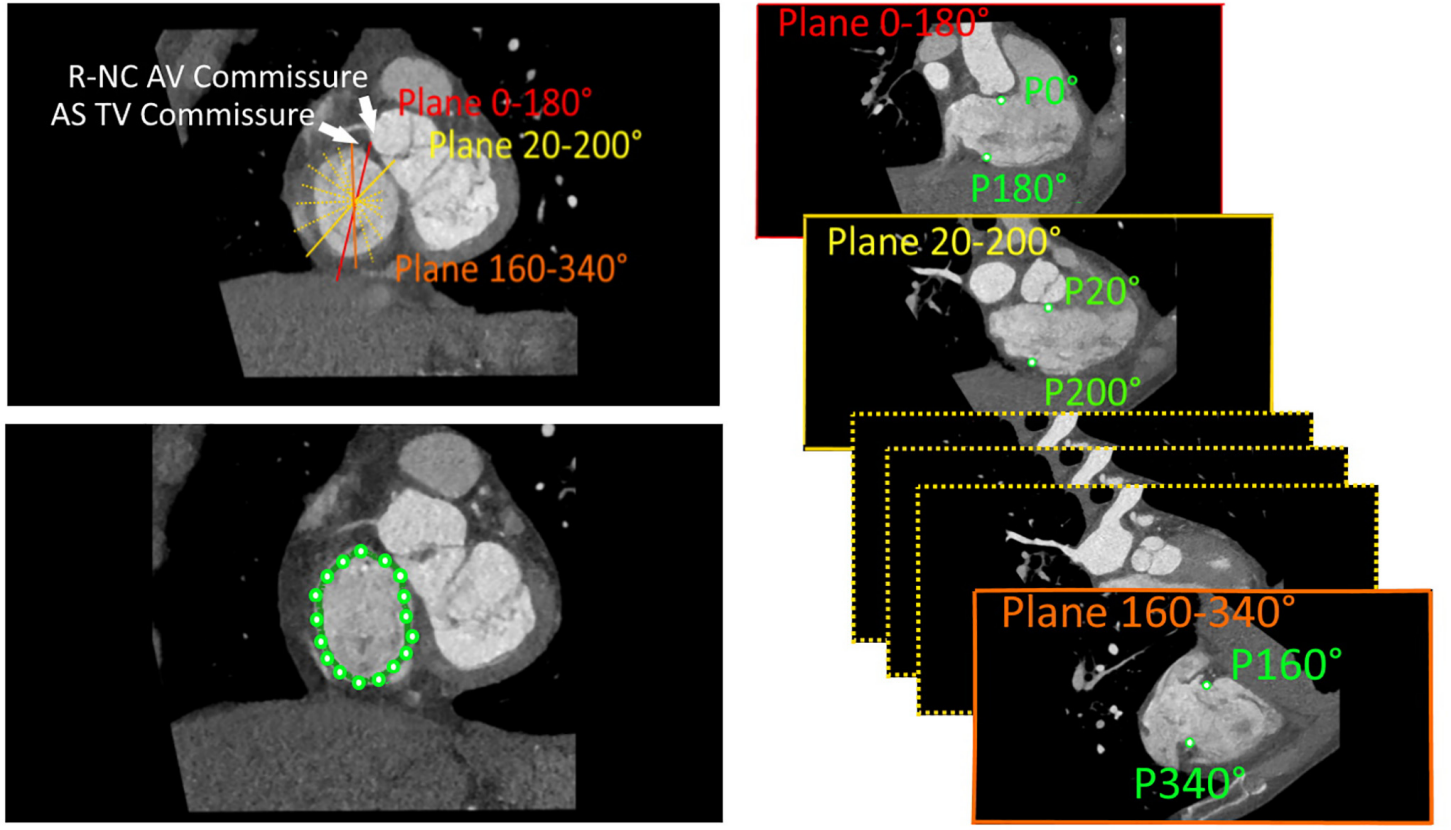
Descriptive representation of the semiautomatic method used to locate the tricuspid annulus (*TA*) at each phase of the cardiac cycle. Multiplanar reconstruction is used to approximatively identify the plane and center of the tricuspid valve (*TV*) orifice. Our custom software generates 9 orthogonal planes that rotate every 20° around the normal axis of the TA approximative plane passing through this center. By convention, the first orthogonal plane (plane 0-180°) is aligned toward the anteroseptal (*AS*) commissure of the TV orifice, which is located just below the right-to-noncoronary (*R-NC*) commissure of the aortic valve (*AV*). Each of these 9 orthogonal planes intercept TA at 2 opposite points, which are pinpointed by the examiner P_i_ and P_i+180°_. The 18 consecutive points of the TV orifice border are interpolated by cubic spline to reconstruct the entire TA.

Measurements of the TA size and shape for each time phase were automated with an in-house software developed at Favaloro University (Lattido). The complete description of the TA reconstruction method using Lattido has been described elsewhere.^14^ In brief, this software displays 9 planes orthogonal to the TA plane in steps of 20° around the normal axis (Figure 1). In each of the 9 planes, 2 seed points were manually pinpointed by the operator in the TA border using the long axis reformatted plane. By convention, the first orthogonal plane (0-180°) was aligned to the commissure between the right and the noncoronary cusps of the aortic valve.

### Best-Fit Plane and Geometric Descriptors

The software used the 3D position of the seeding points to adjust a best-fit plane and to calculate specific geometric features to describe the temporal and spatial change of the TA anatomy in 3D. The best-fit plane was adjusted to the 3D seed points of each time phase using a principal component analysis approach as described before.^14^

In the reference plane, the value of the anteroposterior length between P_0°_ and P_180°_ was called the AP_0-180°_ diameter. For a better description of the elliptical shape of the TA the shortest distance between every pair of opposite points around the TA (ie, $P_{i}$ and $P_{i+180}$ in Figure 1) was adopted as the value of minimal diameter (Min Diameter). The largest distance between each pair of opposing points was taken as the maximum diameter (Max Diameter) and the ratio of the Max Diameter to the diameter orthogonal to it (orthogonal diameter) was used to calculate the eccentricity of the ring using the following formula as:

$$Eccentricity\;Index=1-\frac{Orthogonal\;Diameter}{Maximal\;Diameter}$$

Smaller values of eccentricity representing a more circular TA shape. We also measured the orientation angle of the Max Diameter and Min Diameter at each phase. Angles were expressed between 0° and 180° as explained previously.^14^

In addition, the TA perimeter was divided into 3 segments of 7 contiguous points: septal (P_0°_ to P_120°_), posterior (P_120°_ to P_240°_), and anterior (P_240°_ to P_360°_) for sectorial analysis.

The distance between the 2 farthest centroids of the TA projected in the best-fit planes within the RR interval was calculated as the maximal centroid excursion (MCE) of the TA. Finally, RV contours at ES and ED measured by multiplanar curvilinear reconstruction (MPR) method were used to estimate RV volumes, and RV ejection fraction (RVEF) as the ratio between RV volumes in ED and ES. Using the MPR method, RA contouring was also performed in the middle transverse orthogonal plane to TA at ED and ES to estimate transverse RA area (RA area).

### Statistical Analysis

Analysis was done using IBM SPSS 26.0 (IBM Corp). Continuous variables were reported as mean ± standard deviation, and their normality of distribution was tested using the Shapiro–Wilk test. Shortening was defined as the ratio of maximum and minimum values. All morphometric indices such as diameters, perimeters, and areas were indexed on the body surface area. Comparison of data between groups was performed by a 2-paired *t*-test for continuous variables and χ^2^ or a Fisher exact test for categorical variables. Bonferroni correction was made for multiple testing and the *P* value was adjusted accordingly. Pearson correlation coefficients were used to assess the correlations between 2D and 3D areas and/or perimeters comparisons as relations between diameters, perimeters and areas. Univariate analyses were performed to identify the significant determinants of the maximum 3D area of the TA among clinical, echocardiographic, and CT parameters evaluated by MPR. The most significant variables from this analysis were included in a final model selected to be best associated with the maximum 3D surface of the TA.

## Results

### Baseline Characteristics and Morphometry

Baseline characteristics, echographic and CT morphometric TA parameters, mean values of the study population, and comparisons between groups and subgroups are reported in Table 1. Minimum and maximum values obtained during 1 RR interval of each TA morphometric parameters are presented in Table E1.

**Table 1:** Baseline and echocardiographic and TA morphometric characteristics in each group and each subgroup

	Healthy patients (n = 30)	MR group (n = 21)				DCMP group (n = 21)					
		All MR patients	TR <2+ (n = 12)	TR ≥2+ (n = 9)	*P* vs TR <2+	All DCMP patients	*P* vs MR	TR <2+ (n = 10)	TR ≥2+ (n = 11)	*P* vs TR <2+	*P* vs MR and TR ≥2+
BSA	1.77 ± 0.28	1.79 ± 0.18	1.82 ± 0.16	1.74 ± 0.21	.302	1.97 ± 0.24∗	.007	2.07 ± 0.29∗	1.88 ± 0.15	.070	.081
Age, y	56.4 ± 12.5	52.5 ± 14.9	48.3 ± 14.3	58.2 ± 14.6	.133	52.9 ± 13.0	.930	55.8 ± 13.8	50.3 ± 12.1	.340	.119
Female sex, n (%)	15 (50.0)	11 (52.4)	6 (50.0)	5 (55.6)	<.001	4 (19.0)∗	.052†	2 (20)∗	2 (18.2)∗	.916†	.170
NYHA class	NA	1.8 ± 0.7	1.7 ± 0.6	2.0 ± 0.9	.326	2.9 ± 0.7	<.001	2.8 ± 0.6	3.1 ± 0.7	.032	.006
TTE diameter/BSA, mm/m^2^	17.6 ± 2.7	21.7 ± 4.7∗	20.3 ± 3.0∗	23.7 ± 6.1∗	.113	19.5 ± 4.3	.151	16.7 ± 3.3	22.1 ± 3.5∗	.003	.624
sPAP, mm Hg	30.7 ± 9.4	33.3 ± 9.2	31.6 ± 5.6	35.3 ± 12.3	.428	41.5 ± 13.9∗	.059	43.8 ± 9.6∗	39.8 ± 16.9	.606	.400
3D area/BSA, cm^2^/m^2^	6.2 ± 1.1	8.9 ± 1.8∗	8.2 ± 1.9∗	9.7 ± 1.3∗	.049	8.3 ± 2.6∗	.456	6.5 ± 1.3	10.1 ± 2.4∗	.001	.649
Projected 2D area/BSA, cm^2^/m^2^	6.0 ± 1.1	8.5 ± 1.7∗	7.9 ± 1.8∗	9.3 ± 1.3∗	.042	8.1 ± 2.6∗	.516	6.3 ± 1.3	9.8 ± 2.4∗	.001	.609
3D perimeter/BSA, cm/m^2^	7.0 ± 0.8	8.3 ± 1.1∗	7.9 ± 1.1∗	8.8 ± 1.0∗	.071	7.5 ± 1.4	.062	6.6 ± 0.9	8.4 ± 1.2∗	.002	.486
Septal perimeter/BSA, cm/m^2^	2.1 ± 0.3	2.4 ± 0.4∗	2.3 ± 0.3	2.7 ± 0.4∗	.051	2.2 ± 0.4	.070	1.9 ± 0.3	2.5 ± 0.4∗	.004	.346
Posterior perimeter/BSA, cm/m^2^	2.3 ± 0.3	2.8 ± 0.4∗	2.7 ± 0.4∗	2.9 ± 0.3∗	.105	2.5 ± 0.5	.035	2.1 ± 0.2	2.8 ± 0.4∗	<.001	.460
Anterior perimeter/BSA, cm/m^2^	2.4 ± 0.3	2.8 ± 0.4∗	2.7 ± 0.4∗	2.9 ± 0.4∗	.176	2.6 ± 0.5∗	.201	2.3 ± 0.4	2.9 ± 0.4∗	.003	.936
AP_0-180°_ diameter/BSA, mm/m^2^	21.5 ± 4.1	26.0 ± 4.3∗	24.8 ± 4.7∗	27.5 ± 3.3∗	.161	23.2 ± 4.6	.052	20.2 ± 3.2	26.1 ± 4.0∗	.002	.432
Max diameter/BSA, mm/m^2^	23.4 ± 3.1	28.2 ± 4.2∗	27.1 ± 4.4∗	29.6 ± 3.6∗	.175	25.2 ± 4.4	.031	22.6 ± 3.2	27.7 ± 4.1∗	.006	.297
Max diameter orientation, °	135.3 ± 38.8	128.2 ± 53.4	131.1 ± 49.1	124.3 ± 61.5∗	.782	116.8 ± 61.2	.527	133.2 ± 48.1	100.3 ± 70.6∗	.239	.443
Eccentricity Index	0.58 ± 0.12	0.59 ± 0.12	0.61 ± 0.13	0.57 ± 0.11	.486	0.55 ± 0.11	.212	0.61 ± 0.10	0.48 ± 0.08∗	.004	.048
Min diameter/BSA, mm/m^2^	17.9 ± 2.3	21.0 ± 3.1∗	19.7 ± 2.7∗	22.8 ± 2.7∗	.015	19.9 ± 4.5∗	.354	16.8 ± 2.9	23.0 ± 3.5∗	<.001	.891
Min diameter orientation, °	63.9 ± 43.0	66.3 ± 21.8	60.2 ± 20.8	74.3 ± 21.7	.146	78.2 ± 40.5	.245	79.1 ± 39.8	77.3 ± 43.3	.925	.856

Values are shown as mean ± standard deviation. *MR*, Mitral regurgitation; *DCMP*, dilated cardiomyopathy; *TR*, tricuspid regurgitation; *BSA*, body surface area; *NYHA*, New York Heart Association; *NA*, not applicable; *TTE*, transthoracic echocardiograpy; *sPAP*, systolic pulmonary arterial pressure; *3D*, 3-dimensional; *2D*, 2-dimensional; *AP*_*0-180°*_*diameter*, anteroposterior diameter; *Max diameter*, maximal diameter; *Min diameter*, minimal diameter; *TA*, tricuspid annulus.

∗ Comparison versus healthy: *P* < .05.

† Fisher exact test.

Although 3D and 2D TA areas values were comparable between the MR and DCMP groups, there was a tendency for some parameters such as 3D perimeter, AP_0-180°_ diameter, and Max Diameter to be greater in the MR group compared with the DCMP group (*P* = .061, *P* = .052, and *P* = .031, respectively).

In the MR group, TA area was significantly greater in patients with FTR ≥2+ grades than in patients with FTR <2+ (*P* = .049 and *P* = .042, respectively). However, TTE diameters as well as all other CT diameters were not significant different between these 2 subgroups. In contrast, in the DCMP group, all areas and diameters CT measurements were significantly greater in the subgroup of patients with FTR ≥2+ compared with patients with FTR <2+ (*P* < .01). In addition, only patients with DCMP and FTR ≥2+ had a lower eccentricity index (*P* = .004).

### Dynamics Analysis of the TA

Global fraction of shortening of TA parameters during the cardiac cycle is reported in Table 2. Fraction of shortening of TV orifice 3D area occurring during the cardiac cycle was reduced in both MR and DCMP groups compared with the healthy group (*P* = .050 and *P* = .008, respectively). However, significant alteration of horizontal TA deformations concerned only the first third of the perimeter (P_0°_-P_120°_ septal segment) in patients with MR (*P* = .008), whereas dynamics of almost all 2D parameters were altered for patients with DCMP; notably, fraction of shortening of AP_0-180°_ diameter was reduced in DCMP patients compared with patients with MR (14.4 ± 3.9% vs 18.6 ± 7.0%, *P* = .006).

**Table 2:** RA, RV morphometrics, and TA dynamical parameters in each group and subgroup: The shortenings are the ratio of the difference between the maximum and minimum values to the maximum value (%)

	Healthy patients (n = 30)	MR group (n = 21)				DCMP group (n = 21)					
		All MR patients	TR <2+ (n = 12)	TR ≥2+ (n = 9)	*P* vs TR <2+	All DCMP patients	*P* vs MR	TR < 2+ (n = 10)	TR ≥2+ (n = 11)	*P* vs TR <2+	*P* vs MR and TR ≥2+
Echocardiography											
TAPSE, mm	20.8 ± 3.2	21.5 ± 5.3	22.1 ± 5.3	20.7 ± 5.4	.555	17.6 ± 5.1∗	.020	21.0 ± 4.3	14.7 ± 3.8∗	.003	.015
S-wave, m.s^−1^	12.2 ± 1.6	13.9 ± 2.7	14.2 ± 1.2∗	13.7 ± 3.5	.761	9.5 ± 2.5∗	.001	10.6 ± 2.0	8.5 ± 2.5∗	.074	.005
LVEF	61.0 ± 6.8	60.4 ± 6.8	62.3 ± 5.3	57.9 ± 8.0	.142	28.1 ± 12.3∗	<.001	36.0 ± 11.2∗	21.0 ± 8.5∗	.003	<.001
LV ED diameter, mm	44.7 ± 5.4	56.1 ± 5.3∗	55.7 ± 5.0∗	56.7 ± 6.0∗	.710	64.8 ± 9.9∗	.002	63.1 ± 10.3∗	66.5 ± 9.7∗	.459	.033
LA volume, mL/m^2^	25.0 ± 12.7	58.3 ± 29.0∗	59.5 ± 26.5∗	57.2 ± 32.6∗	.878	46.0 ± 23.4∗	.173	40.2 ± 18.3∗	51.9 ± 27.3∗	.275	.704
RA area, cm^2^	14.9 ± 1.7	15.7 ± 3.5	15.8 ± 3.7	15.5 ± 3.7	.120	22.6 ± 7.1∗	.006	19.3 ± 6.9∗	25.9 ± 6.2∗	.084	.015
Cardiac CT											
TA MCE, mm	−18.1 ± 3.9	−19.6 ± 5.4	−20.5 ± 5.2	−18.4 ± 5.7	.383	−10.5 ± 4.8∗	<.001	−13.3 ± 4.7∗	−7.8 ± 3.0∗	.005	.000
3D area shortening, %	24.1 ± 7.2	20.7 ± 4.9∗	21.9 ± 5.1	19.2 ± 4.5∗	.216	18.4 ± 7.0∗	.219	18.3 ± 6.3∗	18.5 ± 7.9∗	.954	.822
Shortening of septal perimeter, %	27.4 ± 6.7	22.7 ± 4.6∗	23.2 ± 4.5∗	22.0 ± 4.9∗	.576	20.9 ± 7.0∗	.326	21.8 ± 7.7∗	20.0 ± 6.5∗	.589	.454
Shortening of posterior perimeter, %	26.0 ± 5.8	23.2 ± 7.5	23.3 ± 5.1	23.1 ± 10.1	.951	20.6 ± 6.7∗	.255	22.2 ± 7.3	19.1 ± 5.9∗	.303	.301
Shortening of anterior perimeter, %	23.4 ± 6.2	21.3 ± 9.3	20.9 ± 10.4	21.7 ± 8.0	.848	20.5 ± 6.1	.778	18.9 ± 6.4	22.2 ± 5.7	.243	.885
AP_0-180°_ diameter shortening, %	21.0 ± 7.2	19.1 ± 6.0	18.6 ± 7.0	19.8 ± 4.6	.651	14.5 ± 3.9∗	.006	16.2 ± 4.7∗	12.7 ± 1.7∗	.040	.000
RA area ED/BSA, cm^2^/m^2^	9.0 ± 2.2	10.8 ± 4.1	10.0 ± 3.4	11.8 ± 5.0∗	.360	12.8 ± 6.8∗	.238	9.5 ± 4.0	15.9 ± 7.4∗	.025	.169
RA area ES/BSA, cm^2^/m^2^	12.9 ± 2.6	14.6 ± 4.21	14.0 ± 3.8	15.5 ± 4.8∗	.417	14.9 ± 7.1	.871	11.6 ± 4.1	17.9 ± 8.1∗	.040	.444
RV volume MPR ED/BSA, mL/m^2^	78.7 ± 17.4	96.2 ± 23.5∗	92.0 ± 27.6	101.8 ± 16.4∗	.356	120.1 ± 54.9∗	.075	87.6 ± 29.5	152.6 ± 56.0∗	.004	.007
RV volume MPR ES/BSA, mL/m^2^	38.2 ± 12.6	55.5 ± 2.3∗	51.6 ± 16.5∗	60.6 ± 17.9∗	.245	89.7 ± 52.4∗	.007	59.4 ± 30.3∗	119.9 ± 53.4∗	.006	.002
RVEF MPR, %	51.2 ± 8.9	46.6 ± 10.9	51.0 ± 9.5	40.8 ± 10.2∗	.030	28.8 ± 15.4∗	.000	34.1 ± 13.9∗	23.4 ± 15.5∗	.120	.007

*MR*, Mitral regurgitation; *DCMP*, dilated cardiomyopathy; *TR*, tricuspid regurgitation; *TAPSE*, tricuspid annular plane systolic excursion; *LVEF*, left ventricular ejection fraction; *LV ED*, ventricular end diastole; *LA*, left atrium; *RA*, right atrium; *CT*, computed tomography; *TA*, tricuspid annulus; *MCE*, maximal centroid excursion; *3D*, 3-dimensional; *AP*_*0-180°*_*diameter*, anteroposterior diameter; *RV*, right ventricle; *ED*, end diastole; *ES*, end systole; *RVEF*, right ventricular ejection fraction.

∗ Comparison versus healthy: *P* < .05.

**Figure 2:**
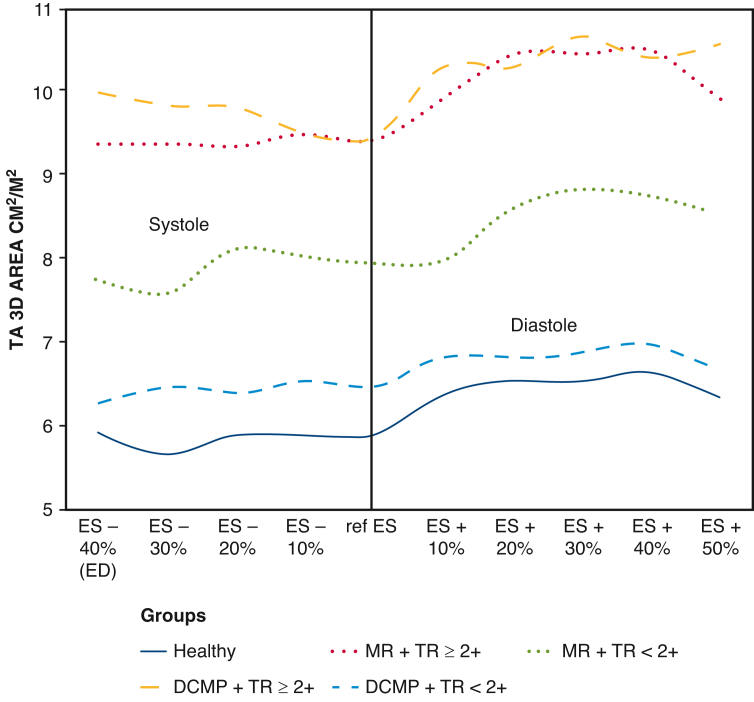
TA 3D areas/BSA according to groups and TR grade during the cardiac cycle. *TA*, Tricuspid annulus; *3D*, 3-dimensional; *ES*, end-systole; *ED*, end-diastole; *MR*, mitral regurgitation; *TR*, tricuspid regurgitation; *DCMP*, dilated cardiomyopathy; *BSA*, body surface area.

**Figure 3:**
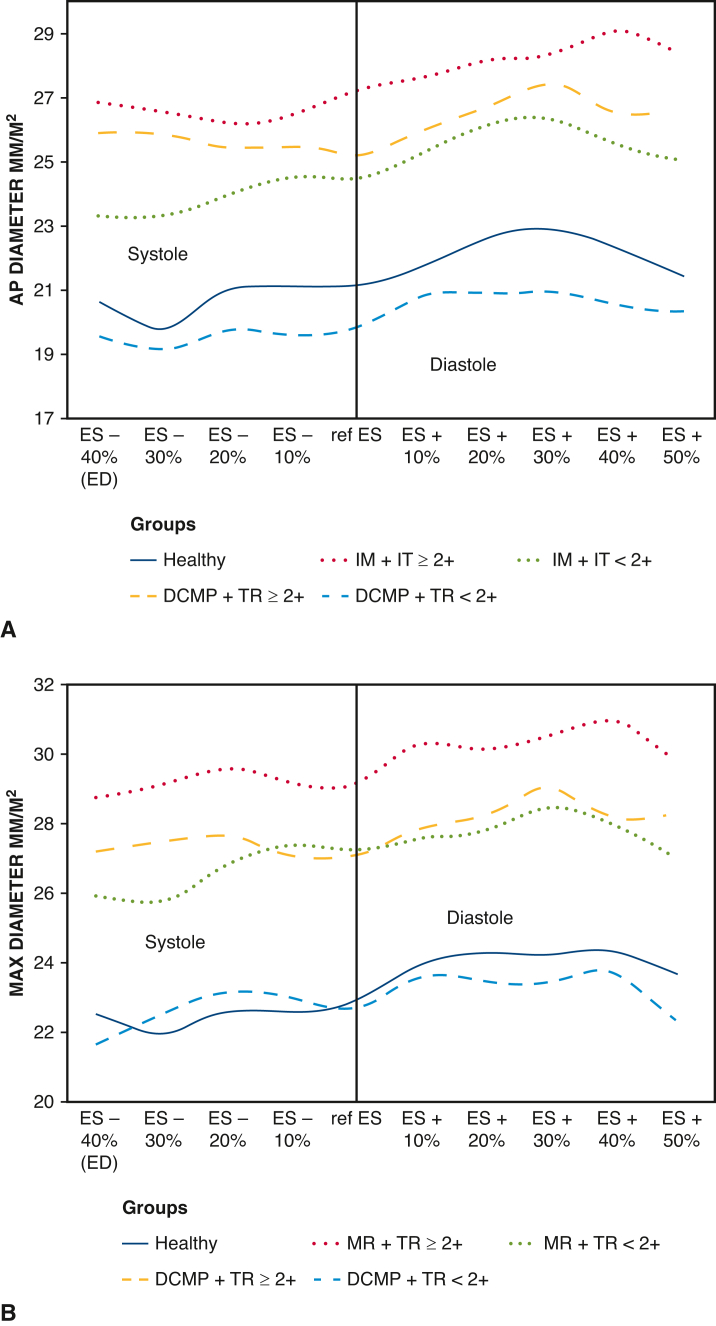
Variation of TA diameters/BSA according to groups and TR during the cardiac cycle. A, AP_0-180°_ diameter; B, Max diameter. *AP*, Anteroposterior diameter; *ES*, end-systole; *ED*, end-diastole; *MR*, mitral regurgitation; *TR*, tricuspid regurgitation; *DCMP*, dilated cardiomyopathy; *MR*, mitral regurgitation; *TR*, tricuspid regurgitation; *TA*, tricuspid annulus; *BSA*, body surface area.

Time analysis revealed that maximal values of 3D parameters occurred in mid-to-late diastole (ES +30% and ES +40%) in all subjects regardless of the presence of tricuspid insufficiency (Figures 2 and 3). Fraction of TA 3D area reduction between ES +30% to ES +40% and ES +50% to ES –40% was 56.4 ± 39.4% for the healthy group and was comparable with pathologic groups 59.4 ± 33.0% and 57.7 ± 35.7% for MR and DCMP groups, respectively, *P* nonsignificant). On the contrary, minimal values were most frequently seen in ES (between ES –20% and Ref ES for patients with TR ≥2+, whereas they occurred in early-systole for patients with FTR <2+ (ES –40% and ES –30%) whatever the group of patients, MR or DCMP.

Morphometric and dynamical values of RA and RV are also presented in Table 2. As expected, compared with the MR group, patients with FTR ≥2+ in the DCMP group had a reduced RV systolic function as characterized by tricuspid annular plane systolic excursion, MCE, and RVEF (*P* = .015, *P* < .001, and *P* = .007, respectively). These patients had also more dilated RA and RV (*P* < .001 and *P* = .003, respectively) and lesser global RA and RV concentric strains compared with FTR <2+ patients with DCMP (*P* = .001 and *P* = .10, respectively). The shortening fraction of TA area was related to both CT RVEF (r = 0.434, *P* < .001) and to systolo-diastolic displacements of the TA directly estimated by MCE (r = 0.327, *P* = .005).

### Predictors of Tricuspid Annular Dilatation

Table 3 shows the results of univariate and multivariate analysis on predictors of maximal value of indexed TA 3D area. ED RA area and ES RV volume were independently associated with TA dilatation (*P* < .001 and *P* = .002, respectively).

**Table 3:** Univariate and multivariate analysis to assess determinants of the maximum 3D area/BSA of the TA

	Univariate analysis		Multivariate analysis		
	Pearson r	*P*	Nonstandard beta- coefficient	SD	*P*
ED RA area/BSA	0.545	<.001	0.198	0.065	.003
ES RV volume/BSA	0.546	<.001	0.033	0.011	.005
RVEF	−0.388	.001	0.048	0.024	.056
Group (healthy, MR, DCMP)	0.408	<.001	0.319	0.388	.414
TR grade	0.419	<.001	0.02	0.286	.944
sPAP	0.32	.007	0.007	0.286	.768
TAPSE	−0.148	.241	–		–
Age	−0.023	.851	–		–
Sex	0.026	.832	–		–
Model coefficient R^2^			0.496		

*SD*, Standard deviation; *ED RA area*, end-diastolic right atrium area; *BSA*, body surface area; *ES RV volume*, end-systolic right ventricle volume; *RVEF*, right ventricular ejection fraction; *MR*, mitral regurgitation; *DCMP*, dilated cardiomyopathy; *TR*, tricuspid regurgitation; *sPAP*, systolic pulmonary arterial pressure; *TAPSE*, tricuspid annular plane systolic excursion; *3D*, 3-dimensional; *TA*, tricuspid annulus.

## DIscussion

TA measurement remains crucial for optimal management of patients who undergo valvular interventions. However, knowledge on human TA morphometry and dynamics under physiological and pathological conditions is lacking to accurately evaluate TA dimensions, and the best imaging method for clinical practice remains to be found. Using the high spatial resolution of cardiac CT and a dedicated software to reconstruct the TA of 3 groups of patients in 3D + time, we demonstrated that (1) TA dimensions were increased in patients with MR due to myxoid mitral valve disease even without significant secondary FTR, (2) the ovoid and saddle shape of the TA is globally maintained in patients with TA enlargement secondary to MR or DCMP, (3) the second half of ventricular filling is the time-phase when TA dimensions are maximal, and (4) ES RV volume and ED RA area are the main determinants of the maximum TA 3D area and independent of each other.

### Morphometry of the TA

The accuracy of cardiac CT measurements in the analysis of TA dilatation in the presence of FTR has already been shown.^16^, ^17^, ^18^, ^19^ Some authors^20^ even found a better correlation between the actual intraoperative TA diameter and its measurement by CT scan rather than 2D TTE. Hassani and colleagues^17^ have also shown that in secondary FTR, even nongated thoracic CT is efficient to measure TA diameter and to detect significant TR. Our results here confirm the small differences observed between the 3D imaging method, which considers the saddle-shaped configuration of the tricuspid annulus and the 2D method, which corresponds to the projection in best-fit plane. This result suggests that 2D planar measurements, which are easily accessible to standard software, will be valid for routine CT TA analysis. Maximal 3D areas values obtained in the present study are thoroughly comparable with those previously reported in ED by Praz and colleagues,^18^ who also used CT scan and a semiautomated method for TA analysis. In the MR group, patients with FTR ≥2+ did not show a significant increase in TTE TA diameter compared with those with FTR <2+, whereas the minimum diameter and more importantly the 2D/3D areas estimated by the cardiac CT were significantly increased. These results call into question the reliability of TTE diameter estimates as a reference method for defining a threshold value of TA dilatation as indicated before mitral valve surgery in recent guidelines.^11^^,^^12^

### Shape of TA

The ovoid shape remained globally preserved in the different groups of our study population. Contrary to the results generally reported in the literature, we did not find that the eccentricity index was significantly modified in our MR group specifically composed of patients with myxomatous disease demonstrating enlarged TV orifice even when FTR <2+. Van Rosendael and colleagues^19^ also reported absence of TA circular remodeling in patients with FTR secondary to aortic valve stenosis undergoing TAVI treatment, although the same authors more recently showed that circular remodeling of the TA is an independent prognostic factor of FTR grade after TAVI treatment.^16^ In patients with DCMP, however, we have observed more circularity on dilated TAs in the presence of FTR ≥2+, since eccentricity was smaller and, although it was not statistically relevant, Max diameter was more septolaterally oriented. Noteworthy, Utsunomiya and colleagues^21^ showed that TA dilatation was more anteroposteriorly oriented in primary FTR compared with secondary FTR. Such data tended to suggest that, within the population of FTR, etiopathogenesis as severity of disease may influence TA shape and size and must be considered when evaluating TA dimensions with 2D imaging methods.

### TA Dynamics

Time analysis also revealed that maximal values of TA morphometric parameters are invariably observed at ES +30% and ES +40%, during the second half of ventricular filling. This temporal aspect needs to be considered in tricuspid orifice maximal measurements, which should focus on the second third of the diastole. Our timing analysis further confirmed that the biphasic pattern of the TA area curve over time, with contraction during systole and expansion during end-diastole, is globally preserved in patients with secondary FTR,^22^ even though TA contraction is globally reduced. Our dynamic analysis also demonstrates that systolic contraction of the TA is delayed at ES between patients with and without FTR, explaining the significant difference observed by Maeaba and colleagues^23^ in the absence of RV dilatation. As it has been shown for the mitral valve, this delay is probably responsible for the onset of TR at the beginning of the RV systole.

### Determinants of TA Size

Our data showed that in sinus-rhythm patients with left-sided heart disease, regardless of FTR grade and sPAP elevation, TA dilatation was independently related to RV dilatation but also to RA dilatation. The fact that RV dilatation is a major determinant of TA dilation was expected in secondary FTR, but the role of RA dilatation requires some argumentation. In the recent concept of atrial “isolated” FTR, without left heart involvement, right atrial dilatation has been associated with annulus dilatation leading to leaflet malcoaptation in systole.^24^ However, in this disease, the role of atrial fibrillation appears to be of major importance and involved in atrial dilatation.^25^ Our study shows for the first time the independent and major relationship of right atrial dilatation with tricuspid annular dilatation in subjects with secondary FTR. Furthermore, we have shown here that the contractility of the TA during the cardiac cycle is related to the contractility of the RV represented by its ejection fraction but, RVEF was found not significantly related to TA dilation in our multivariate model (*P* = .056). Thus, it is therefore important to emphasize here that in patients in sinus rhythm, RV pre-load conditions are more responsible for the TA dilation compared with systolic function.

### Limitations

The main limitation of this study is related to the limited number of patients in each group and overall, in each subgroup. Many comparisons of morphometric parameters between groups are just below statistical significance, which diminishes the power of this study. The time curves presented in this study are derived from mean values of parameters at each time point. The different profiles of each patient were not specifically analyzed and could be very different from the mean values. It should be noted that although we favor cardiac CT over echocardiography, it implies the use of ionizing radiations. However, this modality remains less invasive than coronary angiography and has been increasingly used in the preoperative evaluation of patients undergoing left-sided valve disease with a low probability of having coronary disease. Finally, we have shown that the size of the TA, which is a recognized factor favoring the occurrence of FTR following mitral surgery, is maximal during second half of diastole, ie, during the acquisition period used by the cardiac scanner to correctly visualize the coronary arteries. The work presented here also suggests that 2D or 3D surface measurements of annular size by CT are more satisfactory than diameter measurements for separating patients with or without FTR. The 2D CT area measurement of the TA can be done simply and quickly in less than a minute on a reformatted image by standard software.^16^^,^^19^

## Conclusions

**Figure 4:**
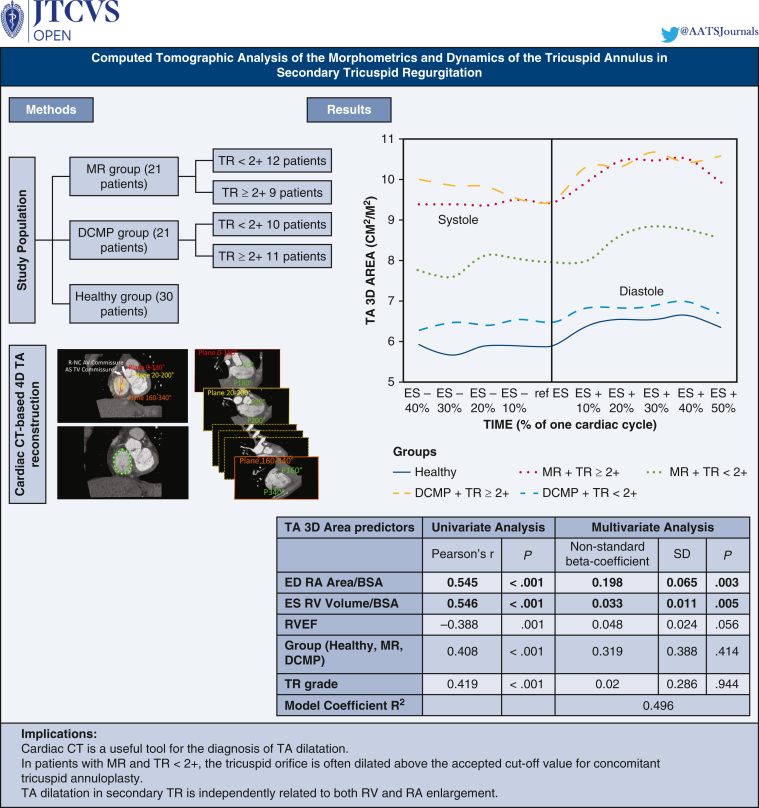
Graphical abstract. *MR*, Mitral regurgitation. *TR*, tricuspid regurgitation; *DCMP*, dilated cardiomyopathy; *CT*, computed tomography; *4D*, 4-dimensional; *TA*, tricuspid annulus; *R-NC*, right-to-noncoronary; *AV*, aortic valve; *AS*, anteroseptal; *TV*, tricuspid valve; *RV*, right ventricle; *RA*, right atrium; *3D*, 3-dimensional; *ES*, end-systole; *SD*, standard deviation; *ED*, end-diastole; *BSA*, body surface area; *RVEF*, right ventricular ejection fraction.

Using the high spatial resolution of multiphase cardiac CT acquisitions, we showed that TA dilatation in secondary FTR is independently correlated to both RA and RV enlargement. In patients with myxomatous mitral valve disease, even in the absence of significant TR, the tricuspid orifice is dilated compared with the standard population and diameters are often above the accepted cut-off value for concomitant tricuspid annuloplasty (Figure 4). By providing accurate measurement of the orifice area and right cavities size, cardiac CT represents an added value to TTE for deciding on preventive intervention on the tricuspid valve during mitral surgery. Further specific clinical studies using cardiac CT will be needed to define the thresholds of abnormal TA surface to predict postoperative FTR after mitral surgery.

### Webcast

You can watch a Webcast of this AATS meeting presentation by going to: https://www.aats.org/resources/finite-computed-tomographic-analysis-of-the-morphometrics-and-dynamics-of-the-right-atrio-ventricular-junction-in-secondary-functional-tricuspid-regurgitation.

**Figure:**
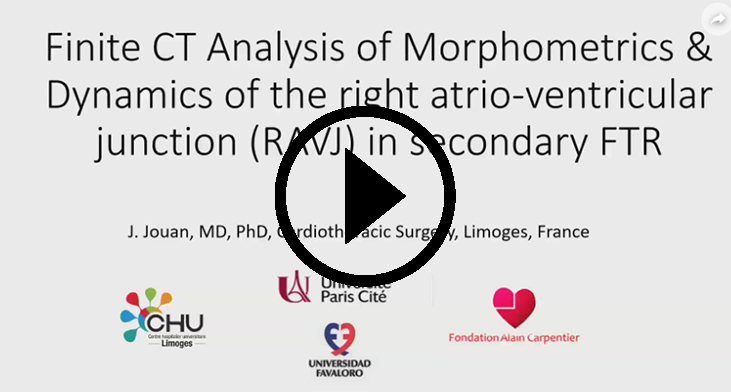

## Conflict of Interest Statement

The authors reported no conflicts of interest.

The *Journal* policy requires editors and reviewers to disclose conflicts of interest and to decline handling or reviewing manuscripts for which they may have a conflict of interest. The editors and reviewers of this article have no conflicts of interest.

## Figures and Tables

**Table E1:** Minimal and maximal values (mean ± SD) of all TA parameters measured at each phase of the cardiac cycle

	Healthy patients (n = 30)	MR group (n = 21)				DCMP group (n = 21)					
		All MR Patients	TR < 2+ (n = 12)	TR ≥ 2+ (n = 9)	*P* vs TR <2+	All DCMP patients	*P* vs all MR	TR <2+ (n = 10)	TR ≥2+ (n = 11)	*P* vs TR <2+	*P* vs MR and TR ≥2+
Area 3D/BSA min, cm^2^/m^2^	5.3 ± 1.0	7.7 ± 1.6∗	7.1 ± 1.6∗	8.6 ± 1.1∗	.023	7.5 ± 2.3∗	.691	6.0 ± 1.3	9.0 ± 2.0∗	.001	.608
Area 3D/BSA max, cm^2^/m^2^	7.0 ± 1.2	9.8 ± 2.1∗	9.1 ± 2.1∗	10.7 ± 1.7∗	.084	9.2 ± 3.0∗	.432	7.2 ± 1.4	11.1 ± 2.8∗	.001	.695
Projected 2D area/BSA min, cm^2^/m^2^	5.1 ± 1.0	7.5 ± 1.5∗	6.9 ± 1.6∗	8.2 ± 1.0∗	.034	7.3 ± 2.3∗	.738	5.7 ± 1.2	8.8 ± 2.0∗	.001	.475
Projected 2D area/BSA max, cm^2^/m^2^	6.8 ± 1.2	9.5 ± 2.0∗	8.8 ± 2.1∗	10.4 ± 1.6∗	.079	8.9 ± 2.9∗	.465	7.0 ± 1.5	10.8 ± 2.8∗	.001	.665
3D perimeter/BSA min, cm/m^2^	6.5 ± 0.8	7.8 ± 1.1∗	7.3 ± 0.98∗	8.3 ± 0.9∗	.029	7.2 ± 1.3∗	.081	6.3 ± 0.9	7.9 ± 1.1∗	.002	.390
3D perimeter/BSA max,cm/m^2^	7.5 ± 0.9	8.8 ± 1.2∗	8.4 ± 1.2∗	9.3 ± 1.1∗	.098	7.9 ± 1.48	.048	7.0 ± 0.9	8.9 ± 1.4∗	.002	.458
Septal perimeter/BSA max, cm/m^2^	2.4 ± 0.3	2.8 ± 0.5∗	2.6 ± 0.4	3.0 ± 0.4∗	.066	2.5 ± 0.5	.036	2.2 ± 0.3	2.7 ± 0.4∗	.006	.209
Septal perimeter/BSA min, cm/m^2^	1.7 ± 0.3	2.2 ± 0.4∗	2.0 ± 0.3∗	2.3 ± 0.3∗	.040	2.0 ± 0.4	.111	1.7 ± 0.3	2.2 ± 0.4∗	.007	.363
Posterior perimeter/BSA max, cm/m^2^	2.7 ± 0.4	3.2 ± 0.6∗	3.0 ± 0.5∗	3.4 ± 0.5∗	.092	2.8 ± 0.5	.031	2.4 ± 0.3	3.1 ± 0.5∗	.003	.260
Posterior perimeter/BSA min, cm/m^2^	2.0 ± 0.3	2.4 ± 0.4∗	2.3 ± 0.3∗	2.6 ± 0.3∗	.054	2.2 ± 0.5	.117	1.9 ± 0.2	2.5 ± 0.4∗	.001	.691
Anterior perimeter/BSA max, cm/m^2^	2.7 ± 0.3	3.1 ± 0.5∗	3.0 ± 0.4∗	3.2 ± 0.5∗	.181	2.9 ± 0.6	.255	2.5 ± 0.4	3.3 ± 0.5∗	.002	.969
Anterior perimeter/BSA min, cm/m^2^	2.1 ± 0.3	2.4 ± 0.4∗	2.3 ± 0.4	2.5 ± 0.4∗	.269	2.3 ± 0.4	.339	2.0 ± 0.3	2.5 ± 0.4∗	.011	.994
AP_0-180°_ diameter/BSA min, mm/m^2^	18.8 ± 4.0	22.9 ± 3.9∗	21.8 ± 4.0∗	24.3 ± 3.4∗	.161	21.4 ± 4.6∗	.261	18.3 ± 3.0	24.4 ± 3.7∗	.001	.917
AP_0-180°_ diameter/BSA max, mm/m^2^	23.7 ± 4.6	28.4 ± 4.9∗	27.0 ± 5.3∗	30.3 ± 3.9∗	.134	24.9 ± 4.9	.030	21.8 ± 3.4	28.0 ± 4.3∗	.002	.253
Max diameter/BSA min, mm/m^2^	21.3 ± 2.8	25.7 ± 3.8∗	24.4 ± 3.7∗	27.5 ± 3.4∗	.070	23.4 ± 4.1∗	.074	20.9 ± 2.9	26.0 ± 3.6∗	.003	.379
Max diameter/BSA max, mm/m^2^	25.4 ± 3.5	30.2 ± 4.7∗	28.9 ± 4.8∗	31.8 ± 4.2∗	.161	26.8 ± 4.7	.026	24.1 ± 3.3	29.4 ± 4.4∗	.007	.245
Eccentricity index min	0.43 ± 0.15	0.45 ± 0.13	0.45 ± 0.13	0.44 ± 0.14	.852	0.42 ± 0.16	.486	0.50 ± 0.13	0.33 ± 0.13∗	.007	.090
Eccentricity index max	0.70 ± 0.10	0.70 ± 0.11	0.70 ± 0.12	0.70 ± 0.09	.966	0.64 ± 0.09	.078	0.70 ± 0.07	0.59 ± 0.07∗	.002	.008
Min diameter/BSA min, mm/m^2^	15.8 ± 2.3	18.6 ± 2.4∗	17.9 ± 2.3∗	19.6 ± 2.3∗	.109	18.4 ± 4.1∗	.875	15.7 ± 2.9	21.2 ± 3.3∗	.001	.226
Min diameter/BSA max, mm/m^2^	19.8 ± 2.6	22.7 ± 3.1∗	21.7 ± 3.3∗	23.9 ± 2.6∗	.113	21.2 ± 4.9	.256	18.0 ± 3.5	24.4 ± 4.0∗	.001	.777

*MR*, Mitral regurgitation; *DCMP*, dilated cardiomyopathy; *TR*, tricuspid regurgitation; *3D*, 3-dimensional; *BSA*, body surface area; *2D*, 2-dimensional; *Max*, maximal; *Min*, minimal; *AP*_*0-180°*_*diameter*, anteroposterior diameter; *SD*, standard deviation; *TA*, tricuspid annulus.

∗ Comparison versus healthy: *P* < .05.
